# Staining of Parasitic Helminths by Extracts of *Allium cepa, Juglans regia*, and *Rubia tinctorum*: An Approach to Herbal Dyes

**Published:** 2018

**Authors:** Zahra MARHABA, Ali HANILOO

**Affiliations:** Dept. of Parasitology and Mycology, School of Medicine, Zanjan University of Medical Sciences, Zanjan, Iran

**Keywords:** Staining, Helminths, *Allium cepa*, *Juglans regia*, *Rubia tinctorum*

## Abstract

**Background:**

Although carmine, as a synthetic dye, is the major substance for staining helminths, it may impose some adverse effects on human health. In the present study, we evaluated the aqueous extracts of onion (*Allium cepa*) skin, walnut (*Juglans regia*) husk, and madder (*Rubia tinctorum*) roots as potential herbal dyes for staining parasitic helminths.

**Methods:**

Aqueous solutions (5%, 10% and 20%, *w/v*) of each herbal prepared from dried and powdered husk of walnut, skins of red onion, and madder roots in distilled water. Parasitic helminths including *Fasciola* spp., *Dicrocoelium denderiticum*, *Echinococcus gronulosus* protoscolices, *Moniezia* spp., and *Haemonchus contortus* were stained by different concentrations of herbal dyes according to carmine staining method. The structural clarity and quality of stained internal organs of the helminths such as suckers, intestine, and reproductive systems were scored by semi-quantitative evaluation in comparison with carmine stained samples.

**Results:**

The optimum concentrations of extracts for helminths staining were 10% (*w/v*) of *A. cepa* and *J. regia*, and 20% (*w/v*) of *R. tinctorum* with final scores of 3.1, 3 and 2.8, respectively. In general, *A. cepa* and *J. regia* extracts showed higher quality in staining Platyhelminthes, while *R. tinctorum* extract presented relatively higher quality in staining Nematoda.

**Conclusion:**

Considering proper quality of *A. cepa*, *J. regia* and *R. tinctorum* extracts in staining the helminths, they may be safe, eco-friendly, and inexpensive alternatives to carmine dye.

## Introduction

Nowadays, carmine compounds as traditional dyes, are the most commonly used dyes for experimental and taxonomic studies of various helminths, particularly parasitic trematodes, cestodes and nematodes (1). Originally, carmine is a natural dye extracted from dried females of an insect named cochineal (*Dactylopius coccus*), commonly used in the cosmetic, pharmaceutical, and food industries. Although this compound properly stains different tissues and organs of helminths, it may impose some adverse effects on human health including risk of dermatologic and respiratory allergies (2, 3). Dyes extracted from plants, animals, and minerals were used as the main color materials in textile, paint, and other industries (4). Afterwards, due to the development of synthetic dyes as cheaper and feasible materials, the use of natural dyes decreased remarkably (5).

Considering worldwide concern and high tendency among researchers favoring the use of eco-friendly and biodegradable sources, the use of natural herbal dyes have come into view once again as an effective alternative. The majority of natural dyes are produced from plant sources such as roots, bark, leaves, wood and other organic sources like fungi and insects. Commercial dyes are more convenient for staining, but herbal dyes may be of higher quality and more appropriate compared to synthetic dyes. In addition, natural herbal dyes are more stable against light, washing and friction (4).

Recently, a number of aqueous and alcoholic extracts of natural dyes such as alizarin, (pigment of madder roots), henna (*Lawsonia inermis*) and curcuma powder (6), sugar beet (*Beta vulgaris*), China rose (*Hibiscus rosasinensis*), and red rose (*Rosa hybrida*) (7) have been evaluated for staining Platyhelminthes. Moreover, extracts of *H. rosasinensis* and *Beta vulgaris* have been employed for detection of some intestinal nematodes eggs (8). These natural extracts have shown the ability to replace the traditional synthetic colors in staining various parts of helminths. However, these studies are preliminary and more researches are required to further evaluate suitability of eco-friendly herbal dyes in staining helminths.

Traditionally, the various parts of walnut tree (*Juglans regia*) and outer skins of edible onion (*Allium cepa*) have been used for dyeing textiles, and recently some experiments also indicated that walnut-based products and onion skin extracts can be used in dyeing cotton, wool and silk (9–12).

In the present study, we evaluated the quality of aqueous extracts of green walnut husk, onion skins, and madder roots (*Rubia tinctorum*) as potential alternative natural dyes for staining some parasitic Platyhelminthes and Nematoda in comparison with commercial carmine dye.

## Materials and Methods

### Preparation of aqueous extracts of herbal dyes

The study was conducted in the Dept. of Parasitology and Mycology, School of Medicine, Zanjan University of Medical Sciences, Zanjan, Iran in 2016–2017.

Dried husk of common walnut (*J. regia*), skins of red onion (*A. cepa*), and dried madder (*R. tinctorum*) roots were ground and powdered by a mechanical grinder. Based on a previous experimental survey on some natural herbal dyes including alizarin (6), 5, 10 and 20 gr of each powder were dissolved in final volume of 100 ml distilled water separately to make the concentrations of 0.05, 0.1 and 0.2 g/ml (5%, 10%, 20%; *w/v*), respectively. The solutions were heated for 3 h at 70 °C in a water bath. After cooling, the extracts were filtered by Whatman filter paper and stored in dark bottles at 4 °C until experiment.

### Sample collection and fixation

The parasitic helminths including *Fasciola* spp. (*F. hepatica* and/or *F. gigantica*), *Dicrocoelium denderiticum*, *Echinococcus gronulosus* protoscolices from infected liver of sheep and cattle, and *Moniezia* spp. (*M. benedeni* and/or *M. expansa*), *Haemonchus contortus* from digestive tract of infected sheep were collected from local slaughterhouse as described previously (13, 14).

After washing by distilled water and relaxation of worms, *Fasciola* spp. and proglottids of *Moniezia* spp. were tied and flattened between two slides (1). All samples were fixed and preserved in 10% formalin until use.

### Staining procedure of the helminths and evaluation

The fixed samples of helminths were stained separately with different concentrations of the prepared herbal extracts (5%, 10% and 20%; *w/v*) as well as carmine dyes (as control) according to common carmine staining method (1) as follows: the fixed samples were washed twice with tap water and immersed in plates of dye solutions (2–6 h) and washed again with tap water. The specimens were transferred into acid alcohol (2 ml of concentrated HCl in 100 ml of 70% ethanol) for 20 sec to 2 min, to remove the excessive stain without loss of pigmentation. Then, samples were subsequently dehydrated in 50%, 70%, 85%, 96%, and 100% ethanol (each step for 15 min), alcohol-xylene (30 min) and xylene (30 min). Finally, they were mounted using canada balsam. The structural brightness and the extent of staining on different parts of the samples such as tegument, hooks, calcareous body, suckers, intestine and reproductive systems (spicule, testes, ootype, vitelline duct, vitellaria, ovary, uterus and eggs) of related helminths were scored by semi-quantitative evaluation on a scale ranging from 1 to 4 points (unstained=1, weak=2, moderate=3 and good=4) by two expert parasitologists independently under light microscopy. For the two independent observations and also in the case of trematodes (*Fasciola* spp. and *D. dendriticum*), the mean scores of similar organs were recorded and analyzed.

The permanent mounted samples monitored microscopically each three months until one year for any possible apparent decrease in quality of staining. However, it was not quantitatively analyzed.

## Results

The aqueous extract of red onion (*A. cepa*) skin appeared dark red in color while aqueous extract of the walnut (*J. regia*) husk appeared brown and madder (*R. tinctorum*) roots extract was brown to yellowish brown in color. The optimum concentrations of different dye extracts for helminths staining were 10% (*w/v*) of *A. cepa* and *J. regia*, and 20% (*w/v*) of *R. tinctorum*, therefore, we showed only the desired concentrations of each dye extracts in Table 1. The internal structures and organs of stained helminths acquired a varying degree of pigmentation using the three herbal extracts which were comparable to carmine stained control samples. Better quality was observed in the samples stained by *A. cepa*, and *J. regia*, with final scores of 3.1 and 3, respectively (Table 1). Concentration of 5% (*w/v*) in none of the three extracted herbal dyes could stain helminths with high quality compared to other concentrations.

**Table 1:** Semi-quantitative evaluation of the stained parasitic helminths using aqueous extracts of herbal dyes; *Allium cepa*, *Juglans regia*, and *Rubia tinctorum*

***Samples of parasitic helminths***	***Staining scores for herbal dyes and carmine***			***Carmine***

	***A. cepa* (mean)**	***J. regia* (mean)**	***R. tinctorum* (mean)**	
*Fasciola* spp. and *D. denderiticum*:				
Tegument and body spines	3.2	3.0	3.3	3
Oral and ventral suckers	4.0	3.5	2.7	3
Intestines	2.5	3.0	2.5	4
Eggs in uterus	3.0	3.0	3.3	3
Ootype and ovary	2.7	2.5	2.3	3
Vitelline glands	3.5	3.0	2.7	3
Testes	3.3	3.0	2.8	3
Total score (mean)	3.2	3.0	2.7	3.1
*Moniezia* spp. and mature proglottides:				
Tegument	3.0	3.0	2.5	3
Ovary and vitelline glands	4.0	2.5	2.5	4
Testes	4.0	4.0	2.5	3
Total score (mean)	3.7	3.2	2.5	3.3
*E. granulosus* protoscolices:				
Suckers	3.0	2.5	3.0	3
Hooks	2.5	2.5	2.5	2
Calcareous bodies	2.5	2.5	2.0	2
Total score (mean)	2.7	2.5	2.5	2.3
*H. contortus*:				
Spicules	3.0	3.0	3.5	4
Ribs	3.0	3.5	3.5	3
Uterus and eggs	3.0	3.0	3.0	3
Intestine	2.5	3.0	3.0	3
Total score (mean)	2.9	3.1	3.3	3.3
Final score (mean)	3.1	3.0	2.8	3.1

**Fig. 1:**
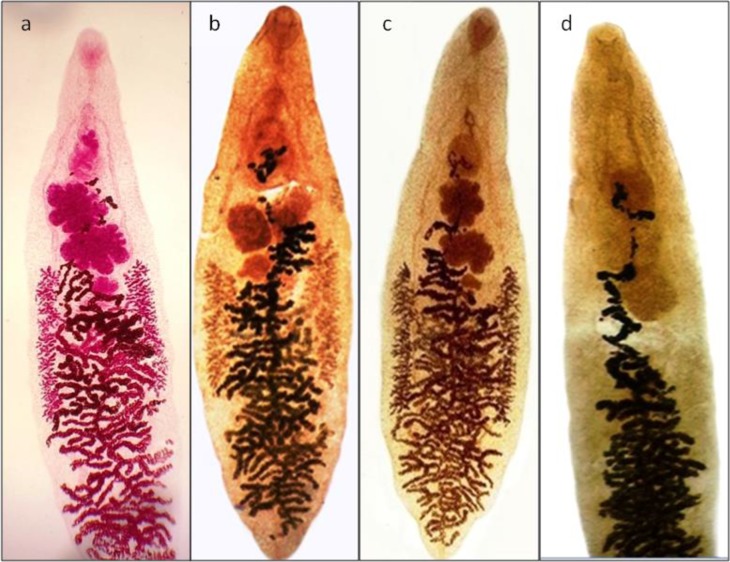
Samples of *Dicrocoelium dendriticum* stained by carmine (a), aqueous extracts of *Allium cepa* (b), *Juglans regia* (c) and *Rubia tinctorum* (d)

**Fig. 2:**
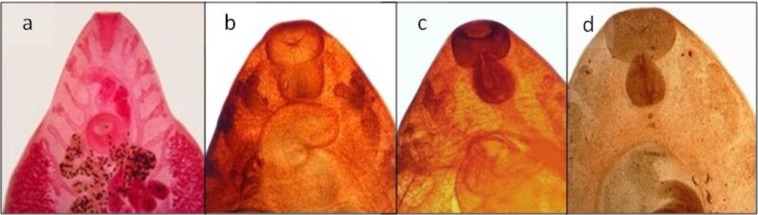
Anterior part of *Fasciola* spp. stained by carmine (a), aqueous extracts of *Allium cepa* (b), *Juglans regia* (c) and *Rubia tinctorum* (d)

Overall, the *A. cepa* and *J. regia* extracts showed higher scores and better quality compared to *R. tinctorum* in staining various organs of trematodes (*Fasciola* spp. and *D. dendriticum*) and cestodes (*Moniezia* spp. proglottids and *E. granulosus* protoscolices) including tegument, suckers, and reproductive system. However, in nematod (*H. contortus*), the *R. tinctorum* extract presented relatively higher quality in the staining of copulatory bursa, female reproductive system, and intestine of the worm (Table 1). Figures 1 and 2 show the stained *D. dendriticum*, and anterior part of *Fasciola* spp. by carmine and optimal concentrations of the three herbal extracts, respectively.

**Fig. 3:**
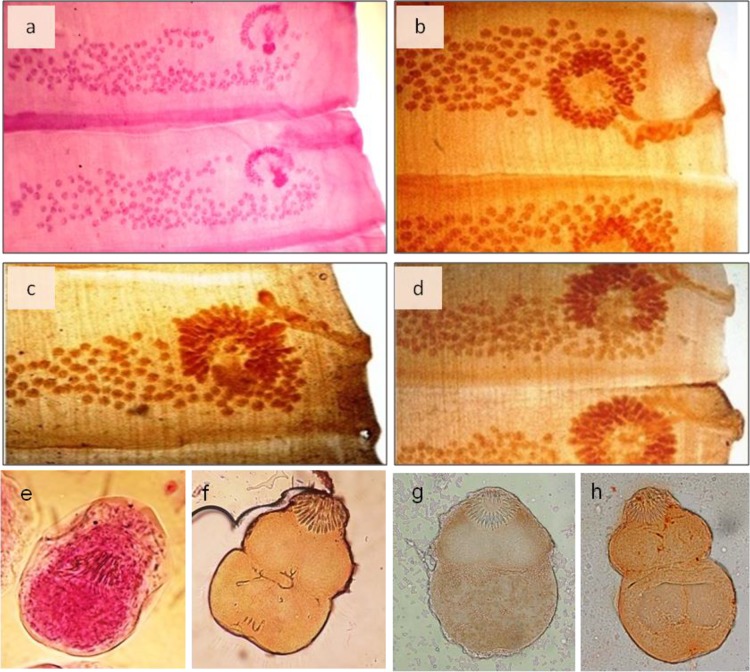
Samples of *Moniezia* spp. proglottids (a-d) and *E. granulosus* protoscolices (e-h) stained by carmine (a, e), aqueous extracts of *Allium cepa* (b, f), *Juglans regia* (c, g) and *Rubia tinctorum* (d, h)

**Fig. 4:**
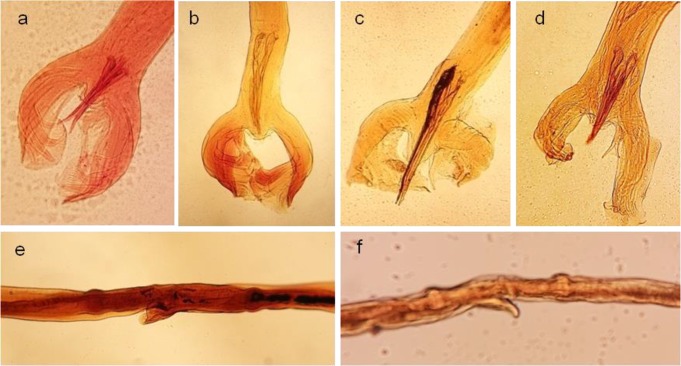
Samples of *H. contortus* copulatory bursa (a-d) and ovijectors (e, f) stained by carmine (a), aqueous extracts of *Allium cepa* (b), *Juglans regia* (c, e) and *Rubia tinctorum* (d, f), respectively

Figure 3 shows the stained proglottids of *Moniezia* spp. and *E. granulosus* protoscolices by the optimal concentrations of the herbal extracts. The stained male copulatory bursa and female reproductive system of the *H. contortus* are presented in Fig. 4.

All three natural herbal dyes could retain the color in permanent mount of the stained helminths for more than 1 year (until drafting this manuscript), without any apparent decrease in quality of staining.

## Discussion

In recent years, employment of eco-friendly and environmentally compatible natural dyes for staining parasites has been one of the major concerns of taxonomists and laboratory technicians to minimize the deleterious effects of the synthetic dyes (6–8, 15). For the first time, we used and evaluated aqueous natural extracts of walnut (*J. regia*) husk*,* onion (*A. cepa*) skin and madder (*R. tinctorum*) roots as alternatives to conventional carmine dye to stain different classes of helminths. Aqueous extracts with concentration of 10% (*w/v*) of *A. cepa* and *J. regia*, and 20% (*w/v*) of *R. tinctorum* have the ability to stain all of the selected parasitic Trematoda, Cestoda, and Nematoda. The qualities of staining by these materials were comparable to carmine-stained samples, however, a few differences were observed in the quality of staining according to type of herbal dye extracts, class of helminths and their internal organs. Although *A. cepa* and *J. regia* extracts showed higher qualit**y** than that of *R. tinctorum* in staining Platyhelminthes including Trematoda and Cestoda, the *R. tinctorum* extract presented higher quality of staining in Nematoda (*Haemonchus*).

Few articles have been published in this field. The effect of aqueous alizarin (the synthetic pigment of *R. tinctorum*), henna (*Lawsonia inermis*), and turmeric (*Curcuma longa*) extracts with different concentrations for the staining of *F. hepatica* were evaluated (6). Alizarin (10 g/100 ml; 10%, *w/v*) was a better dye compared to henna and curcuma for staining suckers, branched intestine, ootype, and vitelline duct. Henna stained the whole body of the worm better than curcuma. However, curcuma showed a better staining result on surface spines, eggs, and particularly branched testes.

Aqueous and alcoholic extract of red beet (*Beta vulgaris*), China rose (*H. rosa-sinensis*), and red rose (*Rosa hybrida*) were used to stain *F. gigantica*, *Gastrothylax crumenifer*, *Taenia solium*, and *M. expansa* (7). The stained Platyhelminthes showed an appropriate level of pigmentation with a proper distinction of internal organs. The suckers and reproductive system of the flukes (*F. gigantica* and *G. crumenifer*) appeared pink with both aqueous and alcoholic extracts of either rose, while the segment of the cestodes appeared pink to red. Either aqueous or ethanolic extracts of China rose and red beet were employed for coloration of *Trichuris* and *Ascaris* eggs, instead of Lugol’s wet mount procedure (8). These extracts were able to stain the nematodes ova in fecal samples. However, in general, ethanol extracts of dyes presented better results comparing to aqueous extracts; additionally, the rose had better quality compared to red beet.

Regardless of the staining quality of the helminths, type of herbal dyes and employed staining protocol, the experiments showed promising and potential ability of the herbal dyes in staining various classes of helminths. However, stability of dyes in the permanent staining of samples is a fundamental issue considered. In the present study, all three natural herbal dyes preserved their dying ability in a dark bottle for about 3 months at 4 °C. Moreover, they could retain their color in the permanent mount of the stained helminths for more than a year (until drafting this manuscript). Such duration of stability has been reported (6) for alizarin, henna and turmeric extracts in stained *F. hepatica* slides. This stability for the rose and red beet extracts in the permanent mount of the Platyhelminthes was more than 6 months (7). In wet mount preparations and clinical diagnosis, long-time stability of the stained samples is not the main parameter. However, in cases such as archiving the samples for future studies and training of students, stability of permanently stained samples could be an important issue. From this point of view, more studies are needed to evaluate the color stability of various herbal dyes.

Herbal dyes are usually eco-friendly and biodegradable materials. Onion plant and walnut are produced in many parts of the world as well-known highly nourishing food sources. Outer onion skin and green walnut husk are inedible and usually discarded as agricultural waste products. Coloring pigment in the onion skin is called pelargonidin (3,5,7,4 tetrahydroxy anthocyanidin), which owing to four hydroxyl groups, displays high-quality dyeing properties for the wool, cotton and silk fabrics (11, 12, 16, 17). The dyeing pigment in the walnut husk is juglone (5-hydroxy-1,4-naphthoquinone) (18). The potential ability of juglone for coloring natural and synthetic fibers including wool, cotton, hair, and polyamide has been studied in detail (9, 10, 18, 19). Additionally, juglone is a molecule with high affinity for bonding with proteins and thus is used to dye skin, hair, fingernails, and leather. The main pigment in the madder roots extract is alizarine (1, 2-dihydroxyanthraquinone), and other anthraquinoid components available in the roots are lucidine and rubiadin which may have carcinogenic properties (18).

## Conclusion

Natural herbal dyes are usually inexpensive, accessible, and eco-friendly materials which make them candidates as proper colorants for staining various parasitic helminths. The aqueous extracts of *A. cepa*, *J. regia*, and *R. tinctorum* have great potential to stain all of the selected parasitic helminths which are comparable to carmine staining. However, a few differences were observed in the staining quality depending on the type of herbal dyes, helminths, and their internal organs. Unlike the madder roots, onion skin and walnut husk extract generally obtained higher scores and better quality in staining Platyhelminthes. The madder roots extracts presented better result in staining nematodes. Finally, we recommend more research to clarify the further dyestuff potential of these herbal extracts in different types of helminths. In addition, optimal extraction methods (aqueous or alcoholic), and concentrations should be further developed and analyzed in further studies.
